# Mid-term follow-up of patients with Brugada syndrome following a cardioverter defibrillator implantation: A single center experience

**Published:** 2007-01-01

**Authors:** A Kharazi, Z Emkanjoo, A Alizadeh, MH Nikoo, MV Jorat, MA Sadr-Ameli

**Affiliations:** From Department of Pacemaker and Electrophysiology, Rajaie Cardiovascular Research and Medical Center, Tehran, IRAN

**Keywords:** Brugada syndrome, Cardioverter defibrillator, Programmed ventricular stimulation

## Abstract

**Background:**

Brugada syndrome is an arrhythmogenic disease characterized by an ECG pattern of ST-segment elevation in the right precordial leads and an increase risk of sudden cardiac death. Risk stratification for the life-threatening arrhythmic events in Brugada syndrome is not yet established. In the present study, we report our experience in patients with Brugada syndrome, following an ICD implantation.

**Methods and Results:**

A total of 12 patients (11 men, 1 woman) with a mean age of 46.5±11.8 were studied. At diagnosis, 7 patients had syncope of unknown origin, 2 patients were asymptomatic, 2 patients were survivors of cardiac arrest, and 1 had documented clinical VT requiring direct cardioversion for termination. Age was similar between the symptomatic and asymptomatic patients (46.6±13 vs. 46±2.8, respectively). Two patients reported a family history of sudden cardiac death. In 3 patients, spontaneous coved-type ECG was found at baseline. In 9 patients, a class I antiarrhythmic drug administration unmasked the characteristic type I ECG. In 4 patients (2 symptomatic with syncope at presentation and 2 asymptomatic), who underwent PES, sustained polymorphic VT or VF was induced. VF was induced by single extrastimuli in 2 symptomatic patients (1 from RV apex and 1 from RVOT). In 2 asymptomatic patients, VF was induced by two and triple ventricular extrastimli (1 from RV apex and 1 from RVOT). None of them experienced an event during follow-up. No significant difference was found between symptomatic and asymptomatic patients (p=NS). The mean follow-up period for the entire study population was 27.83±11.25 months. During follow-up, 2 patients (one with prior cardiac arrest and another with syncope) had VF. Both of them had a type I ECG after provocation with a class I antiarrhythmic drug. None of them had undergone programmed ventricular stimulation. Five patients (41.7 %) had inappropriate ICD interventions during follow-up. The cause of inappropriate therapy was sinus tachycardia in 2 patients, AF in 2 patients and T wave oversensing in 1 patient.

**Conclusion:**

Knowledge about Brugada syndrome is steadily progressing but there are still unanswered issues dealing with the risk stratification and the management of patients.

## Introduction

Brugada syndrome is an arrhythmogenic disease characterized by an ECG pattern of ST-segment elevation in the right precordial leads and increased risk of sudden cardiac death (SCD) as a result of polymorphic ventricular tachycardia or ventricular fibrillation [1].

Studies on the prognosis of patients with asymptomatic Brugada syndrome have reported different incidences of ventricular arrhythmias and SCD, ranging from 0.0% to 8.0% [2-4].  Nevertheless, asymptomatic Brugada syndrome has been elucidated as having a lower risk compared with symptomatic Brugada syndrome.

Although great progress has been made in the identification and characterization of patients with Brugada syndrome, the implantable cardioverter defibrillator (ICD) remains the only proven treatment to prevent sudden death. ICD therapy, however, is not trivial for this frequently young patient population in whom multiple ICD replacements are expected. In addition, for many patients, ICD implantation is not an option because of economic constraints [5,6].

In the present study, we report our experience in patients with Brugada syndrome for whom ICD was implanted.

## Materials and Methods

The study population consisted of 12 patients (11 males, 1 woman) with Brugada syndrome, who underwent ICD implantation in our center. Brugada syndrome was diagnosed after an episode of aborted cardiac arrest, during diagnostic evaluation of syncope of unknown origin or during study of family members of patients with the syndrome, when the following criteria were met: 1) Type 1 ECG pattern as defined by coved-type ST-segment elevation ≥ 2 mm or 0.2mV followed by a negative T wave in the right precordial leads at baseline or after administration of a class I antiarrhythmic drug. 2) the absence of apparent heart disease based on the results of echocardiography, cardiac catheterization, and coronary angiography.

## Electrophysiological study

An Electrophysiologic study (PES) was performed in 4 patients, 2 patients with symptomatic Brugada syndrome and 2 with asymptomatic Brugada syndrome. PES was not performed in 5 symptomatic patients with syncope because they were diagnosed before the report of the second consensus conference by Heart rhythm society and the European heart rhythm association. Programmed ventricular stimulation (PVS) was performed at 2-ms and twice the diastolic threshold current from the right ventricular apex (RVA) and the RVOT, using three basic cycle lengths (600-500 and 400) and a maximum of triple extrastimuli. The endpoints of PVS were either induction of VF or a sustained ventricular tachyarrhythmia (lasting >30 seconds, causing syncope or requiring intervention to be terminated) or completion of PES protocol. No patients received antiarrhythmic drugs before PES.

All patients were followed up at the outpatient clinic. During follow-up, patients were considered to have an arrhythmic event if sudden death occurred or VF was documented in the storage memory of the ICD.

## Statistical Analysis

Continuous variables are expressed as mean ± SD and range and discrete variables are expressed as percentage. Fisher exact test and chi-square test were used to compare categorical variables between groups. A level of p < 0.05 was considered as statistically significant.

## Study limitation

The principle limitation in this study is the small number of patients. The second limitation is mean follow-up of 27.83±11.25 months, which is too short to allow for definitive conclusion about the prognosis of patients with Brugada syndrome.

## Results

Table 1 shows the baseline characteristics of patients, Electrophysiologic study data and events during follow-up. The population consisted of 12 patients (11 men, 1 woman) with a mean age of 46.5±11.8 (median 44 years; 20-68 years). At diagnosis, 7 patients had syncope of unknown origin, 2 patients were asymptomatic, 2 patients were survivors of cardiac arrest, and 1 had documented clinical VT requiring direct cardioversion for termination. Of 2 asymptomatic patients, one was identified during a routine ECG which revealed the characteristic coved-type ECG pattern of Brugada syndrome and another during family screening. Age was similar between the symptomatic and asymptomatic patients (46.6±13 vs. 46±2.8, respectively). Two patients noted a family history of sudden cardiac death, one of them presented with syncope and another had clinical VT associated with hemodynamic collapse. None of them experienced an event during follow-up.

## ECG Parameters and Electrophysiological Study

**Figure 1:**
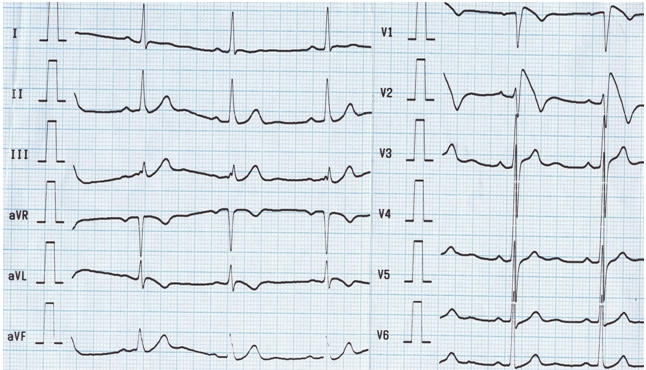
Twelve lead surface ECG showing typical coved-type ST-elevation in right precordial leads

In 3 patients, spontaneous coved-type ECG (type I ECG) was found that was defined at baseline (Figure 1). In 9 patients, a class I antiarrhythmic drug administration unmasked the characteristic type I ECG. In 4 patients (2 symptomatic with syncope at presentation and 2 asymptomatic), who underwent PES, sustained polymorphic VT or VF was induced. VF was induced by single extrastimuli in 2 symptomatic patients (1 from RV apex and 1 from RVOT). In 2 asymptomatic patients, VF was induced by two and triple ventricular extrastimli (1 from RV apex and 1 from RVOT). No significant difference was found between symptomatic and asymptomatic patients (p=NS).

## Therapy and Follow-up

The mean follow-up period for the entire study population was 27.83±11.25 months. During follow-up, 2 patients (one with prior cardiac arrest and another with syncope) had a new arrhythmic event (ICD intervention as a result of VF). Both of them had a type I ECG after provocation with a class I antiarrhythmic drug. None of them had undergone programmed ventricular stimulation. The period from diagnosis to arrhythmic event ranged from 1 to 2 months.

Five patients (41.7 %) had inappropriate ICD interventions during follow-up. The cause of inappropriate therapy was sinus tachycardia in 2 patients, AF in 2 patients and T wave oversensing in 1 patient. In all patients except one who experienced an inappropriate ICD intervention, class I antiarrhythmic drug administration unmasked the diagnostic type I ECG. Three of them had undergone PES and were inducible (1 with single, 1 with double and 1 with triple extrastimuli, respectively).

**Figure 2:**
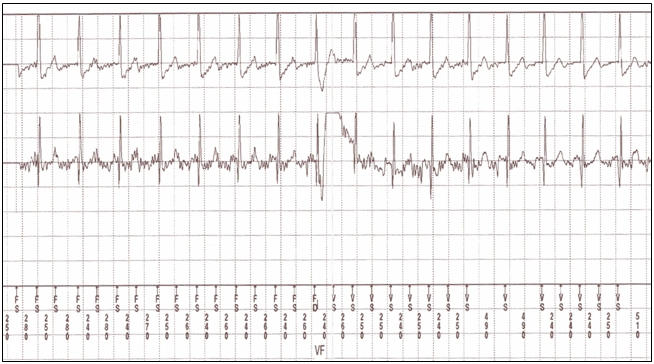
Electrocardiographic recording of oversensing of T wave resulted in the misdiagnosis of sinus tachycardia as ventricular fibrillation

Two patients had a family history of sudden cardiac death, but 3 patients had other family members with Brugada syndrome.  The patient number 2 presented with frequent ICD (Marquis VR 7230, Medtronic Inc., Minneapolis, MN, USA) discharges, two years after implantation. Device interrogation revealed intermittent T wave oversensing due to changes in R:T ratio  which could not be resolved by device reprogramming and required implantation of a new sense /pace lead  in another position where an R wave of greater amplitude made oversensing less likely (Figure 2). In patient number 7, quinidine eliminated repetitive VF episodes, known as electrical storm. Therefore, quinidine was continued in combination with ICD. Neither syncope nor ventricular tachycardia occurred during the administration of quinidine.

## Discussion

Brugada syndrome is an inherited disease that challenges our knowledge. Despite the major advances accomplished during the last decade, there are still unanswered issues dealing with the management of Brugada syndrome. The role of PES for risk stratification is debated. Some investigators supported the view that both symptomatic patients and asymptomatic patients who are inducible by PES are best managed by the implantation of an ICD [7,8]. However, more recent data, supported the view that given the low predictive accuracy of PES, cardiac arrest survivors and patients with a history of syncope or a family history of juvenile SCD should receive an ICD [4,9].

There is consensus that the severity of the clinical manifestation is the most powerful indicator of outcome and those survivors of cardiac arrest and patients with a spontaneous diagnostic pattern with a history of syncope are at higher risk of cardiac events and should receive an ICD without undergoing PES [10]. In our series, 2 patients who experienced an appropriate shock of ICD had a history of syncope and aborted sudden cardiac death. Inappropriate ICD interventions were found in 41.7% of our series during follow-up. Eckardt et al [11], in their study questioned the widespread use of defibrillators in patients with a Brugada ECG but no previous cardiac arrest or syncope. It remains unclear what risk of sudden death is acceptable in a young person. Hence, aggressive management of these individuals may be difficult on the basis of the lethality of the events but has to be weighed against the economic costs and the costs in terms of quality of life, including the risk for inappropriate shocks. In our patients, 2 asymptomatic patients and 2 symptomatic patients who received an ICD based on PES inducibility did not experience an appropriate shock during follow-up.

We showed the high inducibility of VF by less extrastimuli in our symptomatic patients that was concordant with the degree of inducibility reported in previous studies [2,4,9,12].

Marked beneficial effect of quinidine was observed in one of our patients, whose repetitive VF episodes were eliminated. Taking into account the beneficial electrophysiological and clinical effects of quinidine observed in a small number of patients with the Brugada syndrome [12,13], and the antiarrhythmic effectiveness in experimental models [14], further studies of oral quinidine or other Ito blockers in patients with the Brugada syndrome should be encouraged.

The limited follow-up period in our series may explain the lack of events during follow-up. In the presence of low statistical power because of the limited number of events; we could not find the predictors of adverse outcome.

## Conclusion

Knowledge about Brugada syndrome is steadily progressing but there are still controversies about the risk stratification, especially concerning the value of PES. Management of asymptomatic patients is still debated and no conclusive evidence exists to guide risk stratification in this subgroup. A central issue in this study is  inappropriate ICD interventions in 41% of the patients that raise questions about its routine implantation in patients.  The major unresolved question is to determine the criteria for the advisability of ICD implantation.

Appropriate clinical trials are needed to establish the role of antiarrhythmic therapy in selected patient populations with the Brugada syndrome.

## Figures and Tables

**Table 1:**
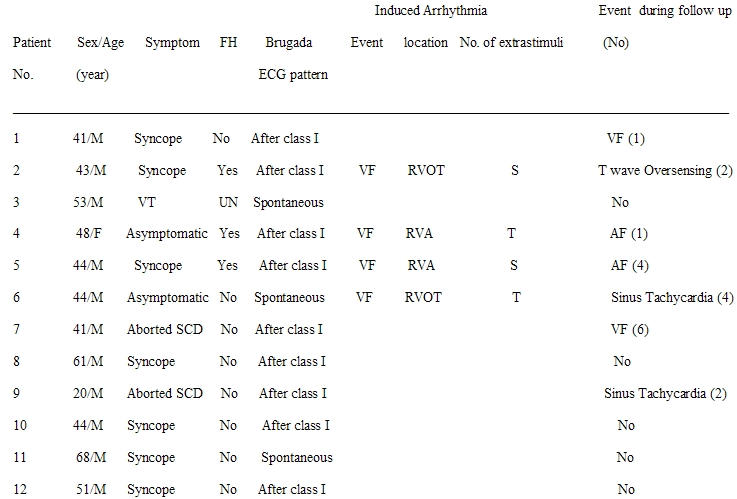
Baseline characteristics of patients

## References

[R1] Brugada P, Brugada J (1992). Right bundle branch block, persistent ST segment elevation and sudden cardiac death: A distinct clinical and electrocardiographic syndrome: A multicenter report. J Am Coll cardiol.

[R2] Brugada J, Brugada R, Antzelevitch C (2002). Long-term follow-up of individuals with the electrocardiographic pattern of right bundle-branch block and ST-segment elevation in precordial leads V1 to V3. Circulation.

[R3] Miyasaka Y, Tsuji H, Yamada K (2001). Prevalence and mortality of the Brugada-type ECGs in one city in Japan. J Am Coll Cardiol.

[R4] Priori SG, Napolitano C, Gasparini M (2000). Clinical and genetic heterogeneity of right bundle branch block and ST-segment elevation syndrome. A prospective evaluation of 52 families. Circulation.

[R5] Priori SG, Aliot E, Blomstrom-Lundqvist C (2001). Task force on sudden cardiac death of the European Society of Cardiology. Eur heart J.

[R6] Belhassen B, Viskin S, Antzelevitch C (2002). The Brugada Syndrome: Is an implantable cardioverter defibrillator the only therapeutic option?. PACE.

[R7] Brugada J, Brugada R, Brugada P (1998). Right bundle branch block and ST-elevation in leads V1 through V3. A marker for sudden death in patients without demonstrated heart disease. Circulation.

[R8] Brugada P, Geelen P, Brugada R (2001). Prognostic value of electrophysiologic investigations in Brugada syndrome. J Cardiovasc Electrophysiol.

[R9] Kanda M, Wataru SH, Matsuo K (2002). Electrophysiologic characteristics and implications of induced ventricular fibrillation in symptomatic patients with Brugada syndrome. J Am Coll Cardiol.

[R10] Priori AG, Napolitano C (2005). Management of patients with Brugada syndrome should not be based on programmed electrical stimulation. Circulation.

[R11] Eckardt L, Probst V, Smits J (2005). Long-term prognosis of individuals with right precordial ST-segment elevation Brugada syndrome. Circulation.

[R12] Belhassen B, Viskin S, Fish R (1999). Effects of electrophysiologic-guided therapy with class 1A antiarrhythmic drugs on the long term outcome of patients with idiopathic ventricular fibrillation with or without the Brugada syndrome. J cardiovasc electrophysiol.

[R13] Watanabe H, Chinushi M, Washizuka T (2005). Variable electrocardiographic effects of short-term quinidine sulphate administration in Brugada syndrome. PACE.

[R14] Antzelevitch C (2001). The Brugada syndrome: Ionic basis and arrhythmia mechanisms. J Cardiovasc electrophysiol.

